# Is Person-Group Value Congruence Always a Good Thing? Values and Well-Being Among Maladjusted Teens and Their Peers

**DOI:** 10.3389/fpsyg.2020.02035

**Published:** 2020-08-25

**Authors:** Agnieszka Bojanowska, Konrad Piotrowski

**Affiliations:** Faculty of Psychology, SWPS University of Social Sciences and Humanities, Warsaw, Poland

**Keywords:** well-being, adolescents, maladjusted behavior, values, congruence

## Abstract

In the present study, we analyzed relationships between values, well-being, and person-group value consistency in two samples: teens under court-mandated supervision (*n* = 51) and teens from the general population (*n* = 49). Results showed that supervised teens experienced lower satisfaction with life, placed more value in stimulation, hedonism, and power, and less in universalism and benevolence. They also experienced lower satisfaction when they valued stimulation, hedonism, and face, and higher satisfaction when they valued conformity-rules and universalism-tolerance. These results show that valuing the things that the group also values at a high level (here: for hedonism and stimulation) may not always be a positive force, especially when the environment is problematic, whereas going against the values of the maladjusted group (here: for universalism) may be beneficial for well-being. However, when we calculated a direct index of person-group congruence, it correlated positively with satisfaction among supervised teens for the values of achievement, stimulation, security-personal, and universalism-concern, whereas congruence for power-dominance correlated with satisfaction negatively among the supervised teens, suggesting a slight but direct limit to the congruence effect.

## Introduction

Research on the relationships between values and well-being has gained popularity since the first publication of Schwartz’s conception of values (Schwartz, 1994). In the first wave of studies, researchers aimed to identify values that facilitated or hampered well-being and indeed found that some values seem to be a bit “healthier,” whereas others are related to lower well-being. On the other hand, the results of their studies yielded inconsistent results—the relationships depended on the sample characteristics, such as the country where the study was conducted or individual characteristics of the research participants (Sortheix and Schwartz, 2017). Naturally, this led to the conclusion that these relationships are moderated by other factors, one of them being the congruence in values between the person and the group. Studies confirmed this relationship, suggesting that a greater congruence between a person and their environment may facilitate well-being because it is easier to realize one’s values when others around us believe in the same things (Sortheix and Lönnqvist, 2015). Is that, however, always true? What if the environment that a person functions in is poorly adjusted, such as in a prison or a work setting that fosters pathological behaviors? In such case, congruence between individual values and group values may have its downsides: it may facilitate engaging in behaviors that are maladaptive, that go against wider social norms or ethics.

## Basic Human Values and Well-Being

One of the most renowned and well-researched conceptions of human values was developed by Schwartz who defines them as “trans-situational goals, varying in importance, that serve as guiding principles in the life of a person or group” (Schwartz et al., 2012, p. 3). They guide human behavior and serve as standards or criteria of what is good or bad, worth doing or avoiding—depending on which values are important for a person (Schwartz, 2012).

In his studies, Schwartz identified a catalog of values that are universal and basic (Schwartz, 2007). According to his model, this catalog forms a circular motivational continuum. Adjacent values can be pursued simultaneously because they share motivational meanings. One of the most recent set of basic values confirmed in intercultural research includes 19 values (Table 1).

**TABLE 1:** Values and their definitions according to Schwartz.

Valued	Definition
Self-direction thought	Freedom to cultivate one’s own ideas and abilities
Self-direction action	Freedom to determine one’s own actions
Stimulation	Excitement, novelty, and challenge in life
Hedonism	Pleasure and sensuous gratification for oneself
Achievement	Personal success through demonstrating competence according to social standards
Power-dominance	Power through exercising control over people
Power-resources	Power through control of material and social resources
Humility	Recognizing one’s insignificance in the larger scheme of things
Conformity-interpersonal	Avoidance of upsetting or harming other people
Conformity-rules	Compliance with rules, laws, and formal obligations
Tradition	Maintaining and preserving cultural, family, or religious traditions
Security-personal	Safety in one’s immediate environment
Security-societal	Safety and stability in the wider society
Face	Security and power through maintaining one’s public image and avoiding humiliation
Universalism-tolerance	Acceptance and understanding of those who are different from oneself
Universalism-nature	Preservation of the natural environment
Universalism-concern	Commitment to equality, justice, and protection for all people
Benevolence-caring	Devotion to the welfare of ingroup members
Benevolence-dependability	Being a reliable and trustworthy member of the ingroup

*Adapted from Cieciuch et al. (2014).*

Initial research on values and well-being suggested that some values are related positively to well-being, whereas others seem unbeneficial in that regard (Sagiv and Schwartz, 2000) because achieving “healthy” values can lead to assessments, attitudes, and behaviors that promote well-being. For example, people for whom benevolence is important think that people are nice, they tend to be tolerant of others and committed to helping them, and these convictions and behaviors would lead to an enhanced well-being. Studies conducted since the 1990s, although there are not many of those, showed that some values are indeed a little “healthier.”

In general, it seems that values such as self-direction, stimulation, and hedonism are related to higher well-being because they express a growth orientation, which motivates people to engage in activities related to self-actualization, the expression of their ideas, abilities, and feelings, and to satisfy the need for autonomy (Yahyagil, 2015; Bojanowska and Piotrowski, 2018; Schwartz and Sortheix, 2018). The function of achievement and power values for well-being is unclear—they also express a growth orientation, but striving for achievement and power may inhibit the ability to maintain positive relationships with other people. A person for whom domination is a key element in their value hierarchy may be difficult in relationships, as cooperation would not be their default stance and they would rather focus on competition. This was confirmed in one study with reference to eudaimonic well-being (Bojanowska and Piotrowski, 2018). The function of humility, conformity, tradition, security, and face values for well-being is unclear. Sortheix and Schwartz (2017) suggest that they express a self-protection orientation (as opposed to growth). They may not be beneficial for well-being because they reflect the need to avoid danger and anxiety that motivate people to submit to the expectations of society to overcome fear. They also express a focus on others (as opposed to personal focus) and therefore address extrinsic needs for status and acceptance. Focusing on others decreases well-being because it directs attention to the needs and problems of others and to social requirements and obligations that limit autonomy. Universalism and benevolence values are consistently positively related to well-being (Cohen and Shamai, 2009; Sortheix and Schwartz, 2017; Bojanowska and Piotrowski, 2018), probably because they foster positive relationships with others and express an underlying positive attitude toward others and a belief that people are good and the world is friendly. Other researchers suggested that the relationship between values and well-being is related to the quiet ego—finding a balance between self-concern and focus on others—which may explain why a combination of universalism, benevolence, and self-direction seem to be beneficial for well-being (Wayment and Bauer, 2018).

The first general hypothesis tested in this study is that some values are positively related to well-being, whereas others are related negatively or are unrelated. We do not formulate a specific hypothesis because the sample is very much different from populations tested in this regard so far. We use satisfaction with life as an index of well-being, as this is one of the most commonly used indices in well-being studies and therefore the data we gather can be compared with others, reported earlier.

As previously mentioned, studies are not conclusive and other reports yielded contradictory results (e.g., Buchanan and Bardi, 2015). Naturally, this led to a conclusion that moderating factors influence relationships between values and well-being. These factors may be sociocultural, such as the level of economic development in the country where the data were collected (Sortheix and Schwartz, 2017), temperament traits (Bojanowska and Piotrowski, 2018), personality (Haslam et al., 2009), or self-esteem (Fetvadjiev and He, 2019). A strong argument has been made that one of the crucial factors moderating these relationships is congruence in values held by individuals and the values preferred in their environments (Sortheix and Lönnqvist, 2015), on which we will focus in this article.

### Person-Group Value Congruence

Maintaining positive relationships with other people in our immediate surroundings is essential to well-being. Good relationships are a source of positive emotions and pleasant experiences. Through these relationships we are able to attain difficult goals that would be unattainable without cooperation with others. They are a source of social support, which has been known to be one of the most stable predictors of individual well-being (Baumeister and Leary, 1995; Helliwell et al., 2016). But how can people foster positive relationships with others in their surroundings? What factors influence and regulate our interactions with others in a way that would ensure smooth cooperation, getting and receiving proper social support, and engaging in exchanges that are pleasant for all involved? One answer to this question would be similarity, which provides more opportunity to express oneself, act according to shared norms, infer correctly about one’s values and reduce dissonance (Biber et al., 2008). People who are similar to one another develop closer bonds; their relationships are more stable and rewarding. This happens both in close, intimate relationships between romantic partners (Gaunt, 2006; Nguyen, 2018) and in bigger groups in, for example, educational environments (Lönnqvist et al., 2009). Similarity may be analyzed in terms of numerous characteristics such as stable traits, for example, temperament or personality, specific interests such as hobbies or work-related activities, or in terms of individually held values. The latter is especially interesting in this context because values determine what people find important and worth pursuing in their lives, determine individual identity, and therefore similarity in values may be especially important in terms of forming relationships with others. It seems natural that people form friendships, choose their work environments or romantic partners basing on their values because discrepancies in values are often the source of conflict and lead to heated debates or arguments that can break down any relationship.

Higher person-group congruence in values can therefore be related to higher well-being because it fosters positive social interactions: people who share the same values communicate better (Edwards and Cable, 2009). This hypothesis was confirmed in a study by Sortheix and Lönnqvist (2015), where the quality of relationships mediated the correlation between value congruence and well-being and partly in a study that showed that the way people form relationships is related to the values they hold (Biber et al., 2008). Also, cooperation based in shared values can lead to better results in goal attainment, which in turn fosters well-being. Studies on the prisoner’s dilemma showed that perceived similarity between partners makes them more eager to cooperate and cooperation is “essential for the success of both individuals and groups. From hunter-gatherer societies to nation-states, the ability of individuals to trust one another and to cooperate is an indicator of prosperity and wealth.” (Fischer, 2009, p. 341). In addition, goal attainment is one of the key elements contributing to satisfaction with life (Tóth et al., 2018). Therefore, a person who functions in a group with similar values would be more eager to assume a cooperative stance. Because of similarity, the partners would then be able to communicate better and achieve their goals and this in turn would impact their satisfaction with life.

### Limits to the Value Congruence and Well-Being Relationship

However, other research did not confirm the robustness of the congruence effect (Kasser and Ahuvia, 2002; Vansteenkiste et al., 2006) and suggested that some values are healthy or unhealthy regardless of their congruence with the environment. There are therefore two hypotheses about the value and well-being relationship: the first suggests a direct effect of specific values on well-being and the other suggests that the function of values depends on the said congruence (Sagiv and Schwartz, 2000). As stated by Joshanloo and Ghaedi (2009), the direct effect of values on well-being has more empirical backing. This makes it even more interesting to analyze whether the congruence effect applies with specific samples not representing the general population, such as samples that are known to have issues with social adjustment. If, for some reason, a person is forced to function in an environment or group that is poorly adjusted, such as a prison, a street gang, or a highly competitive, hostile work environment, we would expect that congruence may be related to poorer well-being or be unrelated at all. These inferences may be drawn from a study by Kasser and Ahuvia (2002) conducted among business students in Singapore, where extrinsic values seem to be encouraged and promoted. Their results contradicted the congruence effect: they showed that holding extrinsic (materialistic) values was related to lower well-being even though they reflected the values of the environment. It is therefore not clear whether finding “partners in crime” in such environment would be beneficial for well-being or whether being similar to other, maladjusted individuals in one’s surrounding could promote pathological behaviors leading to engagement in possibly risky situations. These questions seem even more valid if we consider the period of adolescence, in which peer relationships and peer pressure to behave according to peer norms is very strong and where teenagers are still seeking their own identity and therefore may be more prone to yield to that pressure (Buehler, 2006), and this may lead to value congruence being even more important than among the wider population.

### The Present Study

In the present study, we analyze how individual values are related to teenagers’ well-being, depending on the group that they belong to. We hypothesize that the relationship between values and well-being is moderated by the group that a person belongs to (H1). We do this basing on data from two samples: teenagers under court-mandated supervision, who participate in group educational activities with other teens in a similar situation (attend court-mandated resocialization meetings and extracurricular activities aimed at development of their social skills), and their peers from the general population, matched for basic demographics. The second aim of this study is to show that although most studies suggest that person-group value congruence is beneficial for well-being, there are limits to this effect, and that the level of the group’s general adjustment to social norms is also important. The second hypothesis therefore states that the correlations between congruence in values and satisfaction are more positive and stronger in the general population, compared with supervised teens (H2). The study was approved by the (University name masked for review) Ethics Committee.

## Materials and Methods

### Participants

The participants were *N* = 98 teenagers aged 13 to 18 (*M* = 15.95; *SD* = 1.55) from the two subgroups: court supervised (*n* = 51; 55% women) and community sample (*n* = 47; 45% women). Participants from the first group declared that they had mandated court supervision due to minor problems with the law (minor theft, aggressive behaviors). They attended a re-education center in a mid-sized town in the center of Poland, i.e., participated in classes, workshops (sports, art, basic everyday skills, etc.) but lived at home with their families. The comparison group came from the general population—these teenagers attended public secondary/high schools in the same region as the supervised teens (big town, the schools were neither prestigious nor had a bad reputation). We chose schools that were as close to the supervision centers as possible. Participants were recruited through the re-education center (group 1) and the schools (group 2). After receiving approval from the institution, each participant was approached individually, the aim of the study was explained, and participants were assured that the study was voluntary and their data would be anonymized. Each willing participant received an agreement form, where he/she gave written consent to participate. The participants also had to obtain parental consent (parents also gave written consent on the form). The questionnaires were filled out in a paper-and-pencil format.

### Measures

#### Satisfaction With Life

We used Satisfaction with Life Scale (SWLS; Diener et al., 1985) to assess the global evaluation of life. This measure is an indicator of the cognitive aspect of hedonic well-being (we conceptualized hedonic well-being in accordance with Diener’s subjective well-being approach; Diener, 2000; Kim-Prieto et al., 2005). Individuals with high subjective well-being evaluate their life as positive, according to their own criteria. The measure consists of five items (e.g., “*In most ways, my life is close to my ideal*”) assessed on a scale from 1 = *I definitely disagree* to 7 = *I definitely agree* (score range is between 1 and 7). Higher scores represent higher life satisfaction. Cronbach’s alpha in the sample indicates very good reliability (see Table 2, α = 0.85). The scale also has very good validity confirmed in numerous studies (Pavot and Diener, 2009).

**TABLE 2:** Values and satisfaction with life in the two samples: descriptive statistics and comparison.

				General		
		Supervised		population		
		teens		—teens		Comparison
	Cronbach’s							
	alpha	*M*	*SD*	*M*	*SD*	*F*, *df* = 1,98
AC	0.66	0.47	0.79	0.59	0.75	0.60
HE	0.61	1.11	0.93	0.65	0.79	7.19**
ST	0.62	0.45	1.01	–0.13	0.94	8.74**
SDA	0.71	0.97	0.97	0.71	0.74	2.31
SDT	0.61	0.53	0.78	0.57	0.66	0.10
UNT	0.79	0.06	0.93	0.75	0.84	15.29***
UNN	0.79	–0.44	0.91	0.08	1.16	6.26*
UNC	0.75	–0.18	0.74	–0.68	0.66	12.52**
BEC	0.82	0.62	0.95	0.98	0.60	5.33*
BED	0.74	1.01	0.78	1.17	0.54	1.41
HU	0.62	–0.24	0.97	–0.16	0.75	0.19
COI	0.75	–0.47	1.08	–0.15	0.97	2.38
COR	0.79	–0.59	0.91	–0.40	0.98	0.94
TR	0.84	–0.36	1.02	–0.29	1.08	0.11
SES	0.89	0.38	1.14	0.34	0.90	0.03
SEP	0.64	0.35	0.67	0.24	0.81	0.50
FAC	0.73	0.44	0.94	0.33	0.80	0.43
POR	0.76	–0.29	1.60	–1.13	1.17	8.94**
POD	0.84	–0.68	1.61	–1.89	0.16	19.07***
Satisfaction	0.85	3.84	1.42	4.77	1.04	13.89***

***p < 0.001, **p < 0.01, *p < 0.05. AC, achievement; HE, hedonism; ST, stimulation; SDA, self-direction action; SDT, self-direction thought; UNT, universalism tolerance; UNN, universalism nature; UNC, universalism societal concern; BEC, benevolence-caring; BED, benevolence-dependability; HU, humility; COI, conformity-interpersonal; COR, conformity-rules; TR, tradition; SES, security social; SEP, security personal; FAC, face; POR, power resources; POD, power dominance.

#### Human Values

To measure what participants valued in their lives according to Schwartz’s 19-value model, we used the Portrait of Values Questionnaire (Schwartz et al., 2012). The scale has very good validity, also in the Polish adaptation (Cieciuch, 2013). This questionnaire consists of 57 brief descriptions (or portraits) of different individuals, with three descriptions for each value. Each description portrays a person’s goals and aspirations, introduced with words such as “It is important to him/her,” “He/she thinks,” or “He/she believes.” Example items: *He goes out of his way to be a dependable and trustworthy friend* (benevolence-dependability); *It is important to him to have a good time* (hedonism); *It is important to him to be humble* (humility); *Being very successful is important to him* (achievement). Participants were asked to indicate “How much like you is this person?” using a six-point scale ranging from “Very much like me” to “Not like me at all.” Reliability was satisfactory for all 19 scales. Cronbach’s alphas are shown in Table 2. According to Sagiv and Schwartz (2000), the scores should be ipsatized. First, a sum of points is calculated for each participant and then this sum is subtracted from the sum of points for each subscale. Consequently, scores below zero indicate that the value is relatively less important than the other values, whereas scores above zero indicate that the value is more important.

### Analytical Strategy

Person-group value congruence can be studied subjectively or objectively. In the subjective approach, people are asked how much they feel that their values are compatible with the values in their surroundings (Kristof-Brown et al., 2005), whereas in the objective approach an index of congruence is calculated basing on the average values in the group and individual scores of each participant (Sortheix and Lönnqvist, 2015). We use the objective approach, as it has been underrepresented in hitherto research.

We used two methods of analysis. To verify the first hypothesis, we used regression analysis with the satisfaction with life as the outcome variable and the predictors were values (separately for each of the 19 values) and group assignment (supervised teens versus their peers) and the interaction of value and group. This showed whether a specific value is related to satisfaction with life differently, depending on the group. For the second hypothesis, we used an index of value congruence. It expressed the difference between a teenager’s values and the average weigh of their value in his or her reference group and then squared, so that the index expresses congruence regardless of whether a person’s preference is lower or higher compared with the reference group. This index was inversed, so high values express high congruence.

The formula is as follows:

(Maximal value of difference) - (Person’s value preference - average preference in the reference group)^2^

These two strategies produced a bit different results: the first method provides information on the function of a value depending on the reference group, which may be interpreted as an indirect cue, as to the congruence effect. The second is a more direct way of showing value congruence and its relationship with satisfaction with life (as conceptualized by Diener, 2000).

## Results

We computed descriptive statistics for all the 19 values and satisfaction with life and conducted a one-way ANOVA to compare the two groups. The results of these analyses are presented in Table 2. It turned out that the two groups differed in several values. Teens under court supervision placed lower value in all three types of universalism and in benevolence-caring. In turn, they place higher value in stimulation, hedonism, power-resources, and power-dominance. Teens under supervision had also obtained lower scores on satisfaction with life scale.

In the next step, to analyze relationships between each value and satisfaction with life, Hayes Process macro for SPSS (model 4; Hayes, 2013) was used. In this analysis, the group (supervised teens vs. general population) was introduced as a moderator of the links between each value and life satisfaction to verify whether values relate to life satisfaction similarly in both groups The results are presented in Table 3.

**TABLE 3:** Values, group (supervised vs. general population), and value–group interaction effects for satisfaction with life.

	Satisfaction with life			
Value							
dimension	Value	Group	Interaction	*R*^2^
AC	–0.37	−1.22***	0.52	0.15**
HE	–0.03	–0.09	−0.74**	0.27***
ST	0.09	−0.70**	−0.63**	0.21***
SDA	–0.35	–0.46	–0.39	0.29***
SDT	–0.44	−0.87**	–0.15	0.21***
UNT	–0.15	−1.10***	0.99***	0.30***
UNN	0.08	−0.81*	0.19	0.14**
UNC	–0.19	−0.94**	–0.58	0.22***
BEC	0.42	–0.65	0.42	0.15***
BED	–0.26	–0.66	–0.31	0.18***
HU	0.18	−0.85***	0.28	0.19***
COI	0.16	−0.75**	0.27	0.19***
COR	–0.03	−0.57*	0.62*	0.21***
TR	0.24	−0.81**	0.28	0.24***
SES	0.11	−0.99***	0.16	0.15**
SEP	0.21	−1.03***	0.21	0.16***
FAC	0.21	−0.67*	−0.66*	0.18***
POR	–0.10	−0.95**	–0.32	0.26***
POD	−0.31*	–0.56	–0.03	0.23***

***p < 0.001, **p < 0.01, *p < 0.05. AC, achievement; HE, hedonism; ST, stimulation; SDA, self-direction action; SDT, self-direction thought; UNT, universalism tolerance; UNN, universalism nature; UNC, universalism societal concern; BEC, benevolence-caring; BED, benevolence-dependability; HU, humility; COI, conformity-interpersonal; COR, conformity-rules; TR, tradition; SES, security social; SEP, security personal; FAC, face; POR, power resources; POD, power dominance.

**FIGURE 1:**
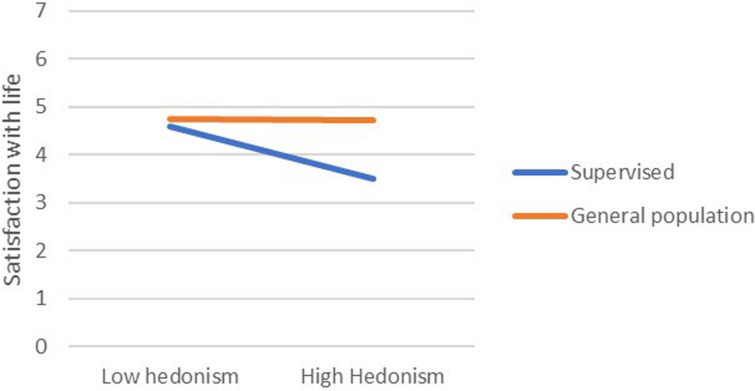
The relationship between Hedonism value and satisfaction with life among teens with court supervision and teens from the general population.

**FIGURE 2:**
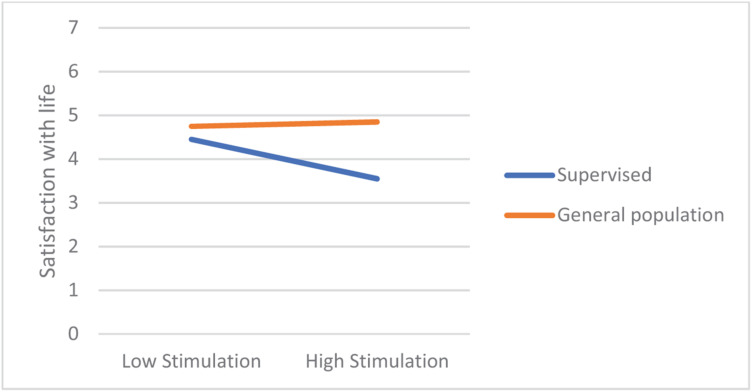
The relationship between Stimulation value and satisfaction with life among teens with court supervision and teens from the general population.

**FIGURE 3:**
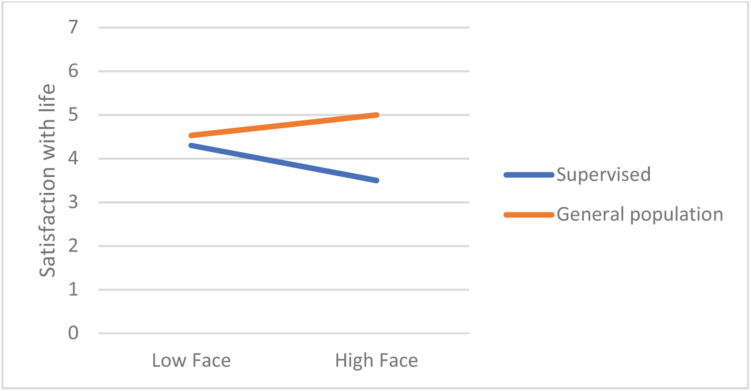
The relationship between Face value and satisfaction with life among teens with court supervision and teens from the general population.

**FIGURE 4:**
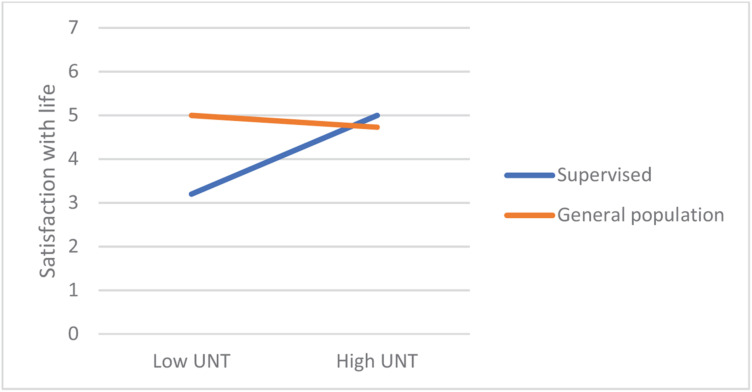
The relationship between Universalism-tolerance value and satisfaction among teens with court supervision and teens from the general population.

**FIGURE 5:**
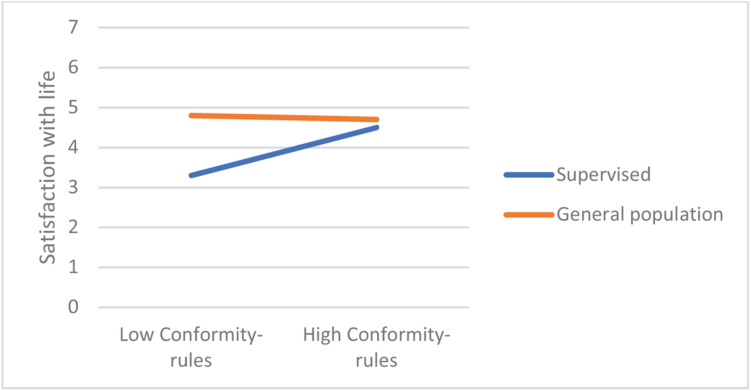
The relationship between Universalism-tolerance value and satisfaction among teens with court supervision and teens from the general population.

In most regression models, being in the supervised group was a significant negative predictor of satisfaction with life. From among the 19 values, only power-dominance value predicted satisfaction (negatively). However, there were numerous significant interaction effects, referring to hedonism, stimulation, universalism-tolerance, conformity-rules, and face. Closer analyses of these interactions revealed that values had small or no effect on the satisfaction in the general population group, but they were moderately related with satisfaction among the supervised teens. In this group, lower satisfaction with life was correlated with more value placed in hedonism, stimulation, and face, whereas satisfaction was higher when they placed higher value in universalism-tolerance and conformity-rules (Figures 1–5). These effects suggest that holding the same values as the poorly adapted group may be related to lower well-being, whereas going against the group may be related to higher well-being.

These interaction effects suggest that the function of the said values is different, depending on the group that the teenager belongs to. However, they do not directly inform about the person-group congruence in values being a serious limitation. To analyze this congruence directly, we correlated the value congruence index (see section “Analytical Strategy”) with satisfaction with life among supervised teens and in the general population subsample and then calculated Fisher’s *Z* to verify if the correlations differ significantly.

The results presented in Table 4 show that the correlations between satisfaction and congruence in achievement, stimulation, security-social, security-personal, and power-dominance differed significantly between the two groups. For achievement, stimulation, security-social, and security-personal congruence, the correlations were stronger among the supervised teens. Contrary to our expectations (H2), the value congruence was related to higher satisfaction especially in the group of supervised teens. However, the effect for power-dominance is consistent with our expectations: higher congruence in this value translated into lower satisfaction with life among the group of supervised teens.

**TABLE 4:** Correlation of values congruence index and satisfaction with life in the two samples: Pearson’s *r* correlation coefficient and Fisher’s *Z* comparison.

	Supervised teens	General population—teens	Comparison
			
	*R* (*n* = 51)	*R* (*n* = 49)	Fisher’s *Z*
AC	0.47***	0.03	2.33**
HE	0.17	–0.01	0.88
ST	0.39**	0.03	1.85*
SDA	0.07	–0.07	0.68
SDT	–0.03	–0.13	0.49
UNT	0.14	0.06	0.39
UNN	0.03	–0.24	1.33
UNC	0.43**	0.21	1.20
BEC	0.22	0.09	0.65
BED	−0.30*	–0.21	–0.47
HU	–0.07	0.09	–0.78
COI	0.20	0.30*	–0.05
COR	0.21	0.29*	–0.41
TR	0.16	0.16	0
SES	0.24*	–0.11	1.72*
SEP	0.39**	0.08	1.61*
FAC	–0.07	0.22	–1.42
POR	0.24	–0.01	1.24
POD	−0.31*	0.09	−1.99*

***p < 0.001, **p < 0.01, *p < 0.05. AC, achievement; HE, hedonism; ST, stimulation; SDA, self-direction action; SDT, self-direction thought; UNT, universalism tolerance; UNN, universalism nature; UNC, universalism societal concern; BEC, benevolence-caring; BED, benevolence-dependability; HU, humility; COI, conformity-interpersonal; COR, conformity-rules; TR, tradition; SES, security social; SEP, security personal; FAC, face; POR, power resources; POD, power dominance.

## Discussion

Values researchers suggest that the link between an individual’s values and subjective well-being may be moderated by other factors and that one of these factors may be the congruence in values between an individual and his/her group of reference (Sortheix and Lönnqvist, 2015). When people that we are close to share a similar values hierarchy, is it easier to get support and obtain a sense of belonging, which results in higher satisfaction with life (Baumeister and Leary, 1995). However, there are also data suggesting that some values are healthier, regardless of the values promoted in the environment (Joshanloo and Ghaedi, 2009). The present study was aimed at explaining the role of values and value congruence for subjective well-being in adolescence. We hypothesized that values play a different role depending on the level of adjustment in the group and that the adaptive function of value congruence could be limited if the social environment that an individual functions in is maladaptive (e.g., street gang or pathological work environment). To verify this suggestion, we studied links between values and well-being in two groups of adolescents: a group recruited from the general population and a group of teens who were under court supervision as a result of breaking the law and who took part in different kinds of resocialization activities.

We found that court-supervised teens had lower well-being, but they also differed in values. They were more oriented on novelty and challenge (stimulation), on feeling pleasure and gratification of their needs (hedonism), and on power. On the other hand, they valued tolerance, natural environment, equality, justice (universalism), and caring for others (benevolence) less than their peers from the general population. Thus, in the group of supervised teens, stronger orientation on individualism and self-development and weaker prosocial orientation can be discerned (Sagiv and Schwartz, 2000) and the value hierarchy may suggest issues with gratification delay that are related also to problematic behaviors (Wulfert et al., 2002)—especially when the hedonism value is considered, which is rated much higher compared with the general subsample from our study (Table 2) and to data presented by other researchers (Cieciuch, 2013).

Our first hypothesis stated that some values would be related to well-being but also that these links will be moderated by group (supervised vs. their peers). First, the results showed that, in general, the relationships between values and well-being are weak to moderate, which is in line with other studies (Vansteenkiste et al., 2006) and with a notion that values are related with eudaimonic rather than with hedonic well-being being studied here (see Joshanloo and Ghaedi, 2009; Bojanowska and Piotrowski, 2018). However, while it was true for adolescents from the general population, values had a significantly stronger impact on supervised teens’ satisfaction with life. Those valuing hedonism, stimulation, or face much, and those who were less oriented on tolerance and conformity had lower well-being than other participants. This observation is in line with the thesis on the moderated impact of values on well-being (Schwartz and Sortheix, 2018). Interestingly, while face and conformity are often related to lower satisfaction with life, hedonism and stimulation usually are related to higher well-being (Sortheix and Schwartz, 2017) because they motivate people to satisfy their intrinsic needs. However, in the group of supervised teens, these values were related to lower well-being. Possibly, it was the social context in which the supervised group functioned during the study responsible for this effect—the supervised teens’ autonomy and self-determination were limited due to court-mandated resocialization activities which could prevent them from satisfying their hedonistic needs (the mediator of the value–satisfaction relationship would therefore need realization). This interpretation is in line with the notion that some values might be adaptive or non-adaptive, depending on the context and a chance to live according to one’s values (Sagiv and Schwartz, 2000). Valuing hedonism and stimulation may be adaptive in the context of free activity when it leads to personal needs fulfillment; however, when an individual’s possibility of self-determination is significantly limited, such as in court-supervised teens that we studied, these values might rather be associated with frustration and anger resulting from externally established limitations preventing from internally motivated activities. It might also explain lower well-being among those supervised teens who valued face much because participating in the resocialization program may be seen by them as a sort of humiliation in the eyes of the other members of the maladjusted group of reference.

Our second hypothesis was that in the adolescent group drawn from the general population, high congruence between individual’s and a group of reference’s values would support well-being, but that in the group of the supervised teens this effect could be limited, i.e., when the reference group held less adaptive values, being highly similar to them might hamper life satisfaction (H2). However, H2 has gained mixed support. In most cases, the results showed that higher value congruence promotes well-being in both subsamples suggesting that high congruence might be universally adaptive. However, in the case of power-dominance in the supervised group, it turned out that indeed, in line with predictions, higher value congruence was negatively correlated with life satisfaction. It should be noted that power-dominance was valued higher by the maladjusted group (Table 2) suggesting that it is highly regarded among court-supervised teens. This result supports our thesis that in some cases, sharing values that are important for the group of reference might lower well-being. Maladjusted teens live in the context of serious deficits in terms of parenting and psychological resources, but also strong hierarchy and rivalry for a position in a group, expressed in “subcultural learning” (Blackman, 2005). When an individual values having control over others, has limited psychological resources, and at the same time is surrounded by people with similar motives, it might lead to more stress and anxiety as an effect of fear over a position in a group. This result, in line with H2, suggests that our hypothesis might be true for some contexts. However, we are aware that this effect may be limited to specific context–values interactions. In the maladjusted adolescents sample, most results were in line with the congruence hypothesis (Sortheix and Lönnqvist, 2015) suggesting that even when an individual is a member of the maladjustment group, similarity of personal and group values hierarchy is associated with higher well-being. These observations turned out to also be true for the general sample adolescents. Still, the effect of power-dominance on court-supervised adolescents that we have observed suggests that the congruence hypothesis can have some limitations associated with the context in which an individual with his/her value hierarchy is embedded. We could recommend intensifying studies on individual-group value congruence in specific, non-mainstream samples as it would help in further developing this field of studies but also in observing its limitations. While past studies suggest that some values are almost universally unhealthy, even when an environment supports them (see studies on external, financial motivation in business students; Kasser and Ahuvia, 2002; Vansteenkiste et al., 2006), our study suggests that in some cases high value congruence between an individual and his/her environment might also be unhealthy, especially when these unhealthy values are highly regarded by the group members.

## Study Limitations and Further Research

While the study shed some new light on the value congruence issue, their results have to be interpreted with caution. First, the number of participants was small, and the sample size may not have enough power to detect the impact of values and value congruence on well-being to a sufficient extent. The subsample of court-supervised adolescents was difficult to reach which resulted in a small sample size; further, we decided to compare them with a general sample similar in size and demographics. This approach, however, allowed us to observe only strong effects with losing some important information. This limitation should be taken into consideration in planning future studies. Second, the study was cross-sectional and their result should be replicated using a longitudinal design. In the discussion section, we suggest that sharing the value of power-dominance with other members of the group might lead to lower well-being. However, to verify this assumption, the court-supervised adolescents should be observed in time with possible reciprocal associations between values and well-being being analyzed. Third, studying such a specific sample significantly limits the possibility of generalization of the results. To verify the presented results and notions, future studies should not only consider bigger sample of court-supervised adolescents but also other specific groups whose value hierarchy could differ from the mainstream, e.g., soldiers, criminals, drug dealers, etc.

## Data Availability Statement

The datasets generated for this study are available on request to the corresponding author.

## Ethics Statement

The studies involving human participants were reviewed and approved by SWPS University of Social Sciences and Humanities Ethics Committee, Faculty in Poznań. Written informed consent to participate in this study was provided by the participants’ legal guardian/next of kin.

## Author Contributions

AB conceptualized the study, prepared the methodology, conducted the analyses, and prepared the initial version of the manuscript. KP verified the analyses and interpreted the results. Both authors contributed to the article and approved the submitted version.

## Conflict of Interest

The authors declare that the research was conducted in the absence of any commercial or financial relationships that could be construed as a potential conflict of interest.
